# Immunological and Preventive Effects of Hochuekkito and Kakkonto Against Coronavirus Disease in Healthcare Workers: A Retrospective Observational Study

**DOI:** 10.3389/fphar.2021.766402

**Published:** 2021-11-18

**Authors:** Keiko Ogawa-Ochiai, Hideki Ishikawa, Hongyang Li, Lam Vu Quang, Izumi Kimoto, Mitsuyuki Takamura, Tetsuya Hongawa, Yasuyuki Hane, Susumu Suzuki, Masaki Okajima, Keita Mori, Masanori Ito, Akiyoshi Takami

**Affiliations:** ^1^ Kampo Clinical Center, Department of General Medicine, Hiroshima University Hospital, Hiroshima, Japan; ^2^ Department of Molecular-Targeting Cancer Prevention, Graduate School of Medical Science, Kyoto Prefectural University of Medicine, Osaka, Japan; ^3^ Department of Internal Medicine, Division of Hematology, Aichi Medical University School of Medicine, Nagakute, Japan; ^4^ Hirose Clinic, Kariya, Japan; ^5^ Kampo Medicine Outpatient Clinic, Mie University Hospital, Tsu, Japan; ^6^ Shizuoka Prefectural Government Office Clinic, Shizuoka, Japan; ^7^ Hane Pediatric Clinic, Toba, Japan; ^8^ Department of Tumor Immunology, Aichi Medical University School of Medicine, Nagakute, Japan; ^9^ Research Creation Support Center, Aichi Medical University, Nagakute, Japan; ^10^ Department of Emergency and Disaster Medicine, Faculty of Medicine Institute of Medical, Pharmaceutical and Health Sciences Kanazawa University, Kanazawa, Japan; ^11^ Clinical Research Support Center, Shizuoka Cancer Center, Shizuoka, Japan; ^12^ Department of General Medicine, Hiroshima University Hospital, Hiroshima, Japan

**Keywords:** Kampo medicine, immunomodulation, COVID-19, SARS-CoV-2, healthcare workers, Kampo formulas, hochuekkito, kakkonto

## Abstract

Amid the global outbreak of coronavirus disease 2019 (COVID-19), it may be expected that low-toxicity natural compounds, such as Kampo formulas, will have a preventive effect on COVID-19. Although the biological properties and safety of the representative Kampo compounds, hochuekkito (HET) and kakkonto (KKT), have been confirmed in various animal model experiments, clinical studies, and a few human studies to induce biological effects on various infectious diseases without significant toxicity, it is unclear whether HET and KKT are safe and effective for COVID-19 prevention. The study population included healthcare workers (HCWs), as they are at a higher risk of infection than the other populations. We retrospectively investigated the immunological and preventive effects of HET and KTT against COVID-19. We included 27 HCWs (aged 21–72 years, F:M = 18:9) from hospitals and clinics of the Hokuriku-Tokai region. The HCWs received HET and KKT for general fatigue and myalgia during this period for 28 days. We obtained patient clinical data from electronic medical records. We analyzed the changes in immunomodulation before and after the administration of the formulas from residual specimens based on the expression of relevant surface markers. The specimens were also tested for the presence of antibodies against severe acute respiratory syndrome coronavirus 2. The following side effects were reported: abdominal discomfort in five patients, diarrhea in two, and loose or soft stool in three. All 27 HCWs tested negative for COVID-19 antibodies. HET and KKT administration significantly increased the absolute number of circulating lymphocytes expressing the activating receptors NKp46, NKp30, and suppressing receptor NKG2A. There was also a significant increase in the absolute number of circulating lymphocytes expressing the receptors TLR4, OX40, 4–1BB, GITR, PD-1, and ICOS. These data indicate that HET and KKT can enhance and modulate NK activity in circulating human immune cells. The immunomodulatory effects, such as activation and regulation of T cells, are consistent with a putative improvement in infectious immunosurveillance. An increase in the number of T cells and CD4/CD8-positive cells indicates an enhanced ability to protect against infection. HET and KKT may prevent the onset or worsening of COVID-19 through their immunomodulatory effects.

## Introduction

Healthcare workers (HCWs) may experience an increased risk of severe acute respiratory syndrome coronavirus-2 (SARS-CoV-2) infection because of close contact with infected patients. HCWs have emerged as a critical population during the current coronavirus disease (COVID-19) pandemic. During the pandemic, 138 patients, including 40 HCWs (29%), were admitted to Zhongnan Hospital in Wuhan (Wang et al., 2020). Another retrospective analysis of 1099 patients with confirmed COVID-19 in 552 hospitals from 31 provinces found that the proportion of HCWs was 2.09%. It is very important to safely protect frontline HCWs from acquiring severe SARS-CoV-2 infections. The plight of HCWs during the pandemic has been widely noted (Gao et al., 2020; Kluytmans-van den Bergh et al., 2020). Moreover, infection control requires a lot of effort and time, and it is presumed that most HCWs become exhausted and reach an immunocompromised state. Between February and July 2020, hochuekkito (HET) and kakkonto (KKT) were prescribed to patients who wanted the HCWs to alleviate fatigue.

Hochuekkito (HET, Bu-Zhong-Yi-Qi-Tang in Chinese) is an herbal formula of Kampo medicine that is widely used for the treatment of severe weakness, loss of appetite, and indigestion in elderly patients and for the prevention of opportunistic infections. HET contains the following 10 crude drugs: Astragali radix (*Astragalus membranaceus* var. *mongholicus* (Bunge)), Atractylodis lanceae rhizoma (*Atractylodes lancea* (Thunb.) DC.), Ginseng radix (*Panax ginseng* C. A. Mey.), Angelicase radix (*Angelica acutiloba* (Siebold and Zucc.) Kitag.), Bupleuri radix (*Bupleurum falcatum* L.), Zizyphi fructus (*Ziziphus jujuba* var. inermis (Bunge) Rehder), Citri Unshiu Pericarpium (*Citrus unshiu* (Yu.Tanaka ex Swingle) Marcow.), Glycyrrhizae radix (*Glycyrrhiza uralensis* Fisch.), Cimicifugae rhizoma (*Betula dahurica* var. maximowiczii (Rupr.) Trautv.), and Zingiberis rhizome (*Zingiber officinale Roscoe*) The therapeutic effects of HET have been reported in terms of improving weakness in the elderly (Kuroiwa et al., 2004; Satoh et al., 2005) and preventing chronic obstructive pulmonary disease in the elderly (Tatsumi et al., 2009). HET has demonstrated improvements in fungal infection (Abe et al., 1999), protective action against *Listeria monocytogenes* infection (Yamaoka et al., 2001), and antiviral action through the activation of natural killer (NK) T cells (Yamaya M, 2007). HET is expected to exert effectiveness for preventing infectious diseases including COVID-19. HET promotes the production of interferon and inhibits the production of interleukin (IL)-1α and IL-6 (Tokura et al., 1998; Mori et al., 1999). In this study, HET was used in the form of spray-dried decoction extracts of a mixture of ten medicinal plants (manufactured by Tsumura and Co., Tokyo, Japan).

Kakkonto (KKT), a traditional Japanese herbal medicine, is commonly used in Japan. KKT contains the following seven crude drugs: Cinnamomi cortex (*Cinnamomum cassia* (L.) J. Presl), Zingiberis rhizome (*Zingiber officinale Roscoe*), Paeonia lactiflora Radix (*Paeonia lactiflora Pallas*), Zizyphi fructus (*Ziziphus jujuba* var. inermis (Bunge) Rehder), Glycyrrhizae radix (*Glycyrrhiza uralensis* Fisch.), Ephedrae Herba (*Ephedra sinica* Stapf) and Puerariae Radix (*Pueraria montana* var. lobata (Willd.) Maesen and S.M.Almeida ex Sanjappa and Predeep). KKT is considered to be a highly effective and safe medicine for the treatment of the common cold or influenza in Japan; the Ephedra herb in KKT contains tannins, and they inhibit endosome acidification and influenza A virus fusion to the cell membrane (Mantani et al., 1999). Glycyrrhizin, an active component of glycyrrhiza, exerts its effects by reducing the number of cells infected with the influenza A virus and by inhibiting viral uptake through the cell membrane during the early phase of infection (Wolkerstorfer et al., 2009). Cinnamaldehyde, which is derived from cinnamon bark, inhibits viral protein synthesis (influenza A virus) at the post-transcriptional level. In one research carried out in mice, inhalation and nasal inoculation of cinnamaldehyde after viral infection increased the survival rate (Hayashi et al., 2007). Puerarin, one of the bioactive compounds from Puerariae Radix, is an isoflavonoid that exerts many pharmacological effects such as anti-inflammatory, vasodilation, neuroprotective, antioxidant, and anticancer effects (Zhou et al., 2013). Kampo formulas including HET have been approved by the Ministry of Health, Labor, and Welfare in Japan as a prescription covered under the National Health Insurance; therefore, HET can be easily used for the treatment of patients.

In medical literature, there is accumulating evidence for the biological properties and safety of HET and KKT, but it is unclear whether HET and KKT are safe and effective for COVID-19 prevention. In this study, we intended to study the effectiveness of the HET/KKT combination in improving immunity, which explains the empirical use of these formulas based on Kampo diagnosis to clarify the clinical indications of HET for the prevention of infectious diseases.

Therefore, we aimed to retrospectively determine the risk and preventive effects of HET and KKT on COVID-19 and identify immune response features through immunological investigation. We conducted this study in HCWs who received HET and KKT.

## Materials and Methods

### Study Design

A total of 27 HCWs (male, *n* = 9; female, *n* = 18; median age, 48 years (range, 21–72 years)] were enrolled in this retrospective observational study, which was approved by the Kanazawa University Review Board (approval no. 2020-015) and conducted in accordance with the Declaration of Helsinki. All subjects provided written informed consent to participate in this study. The inclusion criteria were as follows: HCWs receiving HET and KKT daily (5–7.5 g/ day, twice or three times a day before meals, Tsumura, Tokyo, Japan) with standard insurance coverage as patients, those whose blood samples were collected before and approximately 14 or 28 days after taking the Kampo formulas. In clinical practice, HET is often used for fatigue. On the other hand, KKT is used for musculoskeletal pains that HCWs often complain of, such as stiff shoulders, and is often used in combination with HET in actual clinical practice. In this study, many HCWs were prescribed these two prescriptions together because the number of HCWs complaining of fatigue due to the increased workload in the medical field following the COVID-19 pandemic.

The primary outcome of this study was to assess the potential effects of HET and KKT on circulating lymphocytes. The secondary outcome was to evaluate the safety of HET and KKT. In Kampo Clinic, patients are usually evaluated around 2 weeks to 1 month after the first administration of Kampo formulas to investigate their drug compliance and the incidence of adverse events; therefore, we retrospectively evaluated the patients using their charts and laboratory data.

Since there were no cases in the group of cases evaluated that developed symptoms during the course of the disease, no PCR or other tests were performed, and antibody measurement was performed on the serum after 1 month by HISCLTM SARS-CoV-2 N-immunoglobulin G (N-IgG), N-IgM, S-IgG, and S-IgM (Sysmecs, Kobe, Japan).

### Sample Preparation and Pharmacokinetic Evaluations

Residual blood samples with EDTA-2Na were centrifuged under refrigeration (for 8 min, 3,000 × *g*) to isolate serum. Peripheral blood mononuclear cells (PBMCs) were isolated from the remaining blood by gradient centrifugation. A fraction of the isolated PBMCs from each sample was cryopreserved for further use.

### Whole-Blood Cell Counts

Whole-blood cell counts and individual leukocyte fractions were analyzed individually at each hospital or clinic. Absolute cell counts were calculated by multiplying the leukocyte count.

### Flow Cytometry

Isolated PBMCs were stained with antibodies specific to the cell surface markers of NK lymphocytes, T cells, and B cells, including anti-CD3, CD19, CD20, CD4, CD8, CD16, CD56, NKp46, NKG2D, NKp30, TLR4, DNAM-1, NKG2A, 4-1BB, OX40, ICOS, PD-1, CTLA4, GITR, LAG3, TIGIT, and TIM3 (BioLegend, San Diego, CA, United States). The stained cells were analyzed using FACSCant II (BD Biosciences, San Jose, CA, United States), and the data were analyzed using the FlowJo software package (ver. 10; Tree Star, Ashland, OR, United States). In the PBMC gating, CD3^+^CD56^−^cells were defined as T cells, CD3^−^CD56 ^+^ cells were defined as NK cells, CD19 + CD20 ^+^ cells were defined as B cells, and T cells were further divided into CD4^+^ T cells and CD8^+^ cells.

### Statistical Analyses

Data are reported as median ± minimum and maximum values. When comparisons were made between two different groups, statistical significance was determined using Wilcoxon signed-rank test. Statistical significance was set at *p* < 0.05.

## Results

### Safety and Compliance of the Kampo Formulas

Twenty-seven subjects who were enrolled in this study received 2-3 packs of HET and KKT daily (5–7.5 g/ day, Tsumura, Tokyo, Japan) for more than 28 consecutive days. All subjects had more than 70% adherence rate for Kampo formulas.

Abdominal discomfort was found in five (3 females, 2 males), diarrhea in two (1 female and 1 male), and loose or soft stool in three (all of them are females) patients. No other serious side effects were observed.

### Effects of HET and KKT on Complete-Blood Cell Counts

**FIGURE 1:**
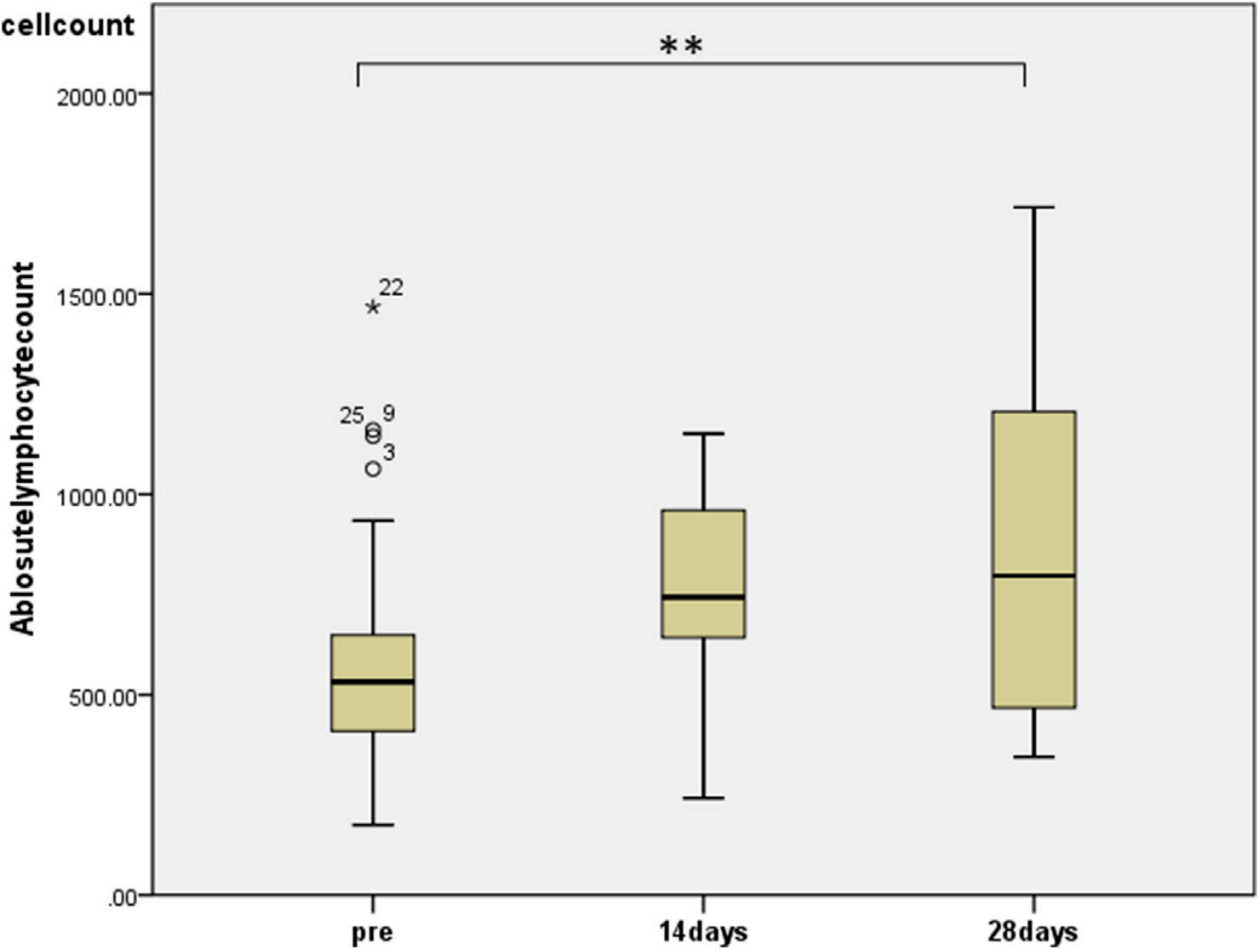
The effects of HET and KKT on whole blood cell counts. HET and KKT administration resulted in a significant increase in the absolute lymphocyte count in the blood.

To assess the effects of the administration of KKT and HET in HCWs, we first performed whole-blood cell counts at baseline and after the administration of KKT and HET using a blood cell counter. We observed significant changes in the number of lymphocytes (median 532, range 175–1,145 before administration; median 797, range 345–1716 at day 28, *p* < 0.01) in the blood (Figure 1).

### Effects of HET and KKT on Circulating PBMCs

**FIGURE 2:**
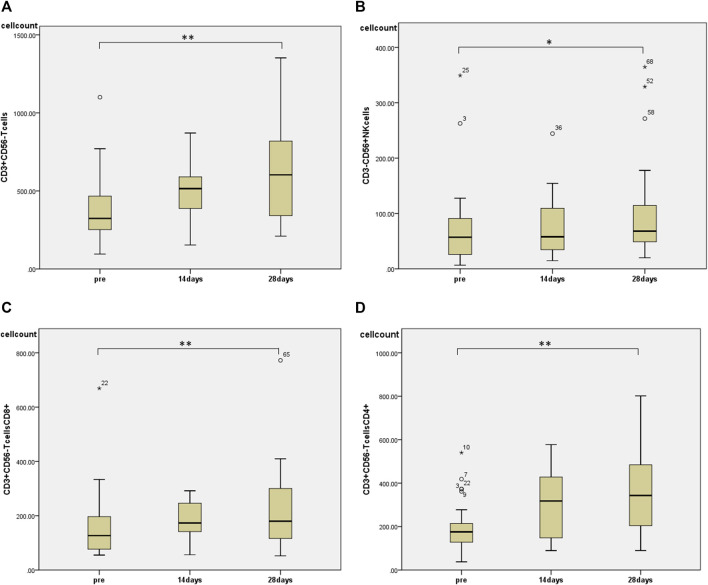
The effects of HET and KKT on circulating peripheral blood mononuclear cells (PBMCs). The administration of HET and KKT resulted in a significant increase in the **(A)** T cell population; **(B)** NK cells; **(C)** CD_8_^+^T cells, and **(D)** CD_4_^+^ T cells. * *p* < 0.05, ** *p* < 0.01.

**FIGURE 3:**
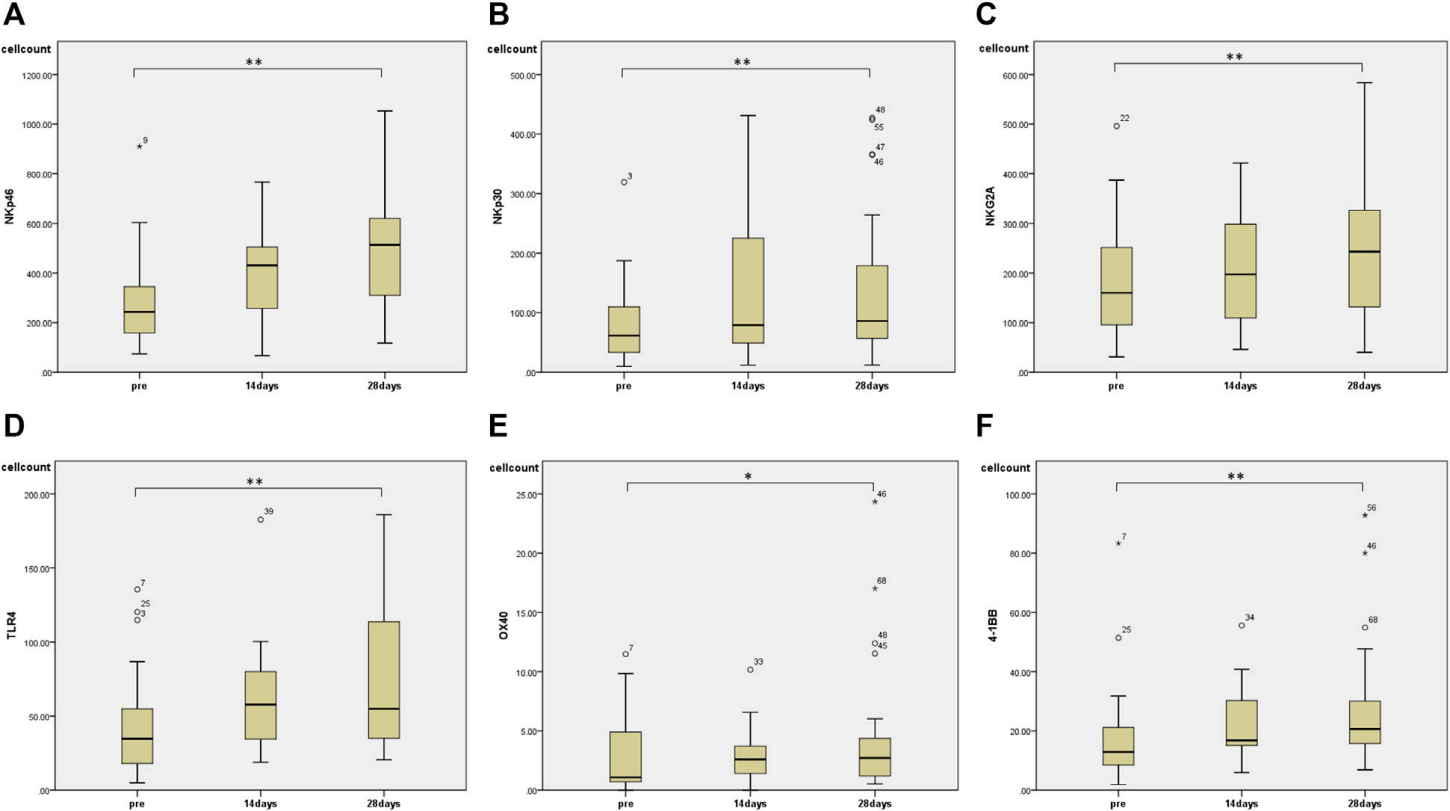
The effects of HET and KKT on PBMC subsets. The 28 days administration of HET and KKT resulted in a significant increase in the **(A)** activator receptors NKp46 **(B)** NKp30, and **(C)** suppressing receptor NKG2A. Expression of other cell surface markers including **(D)** TLR4; **(E)** OX40, and **(F)** 4-1BB also significantly increased. * *p* < 0.05; ** *p* < 0.01.

**FIGURE 4:**
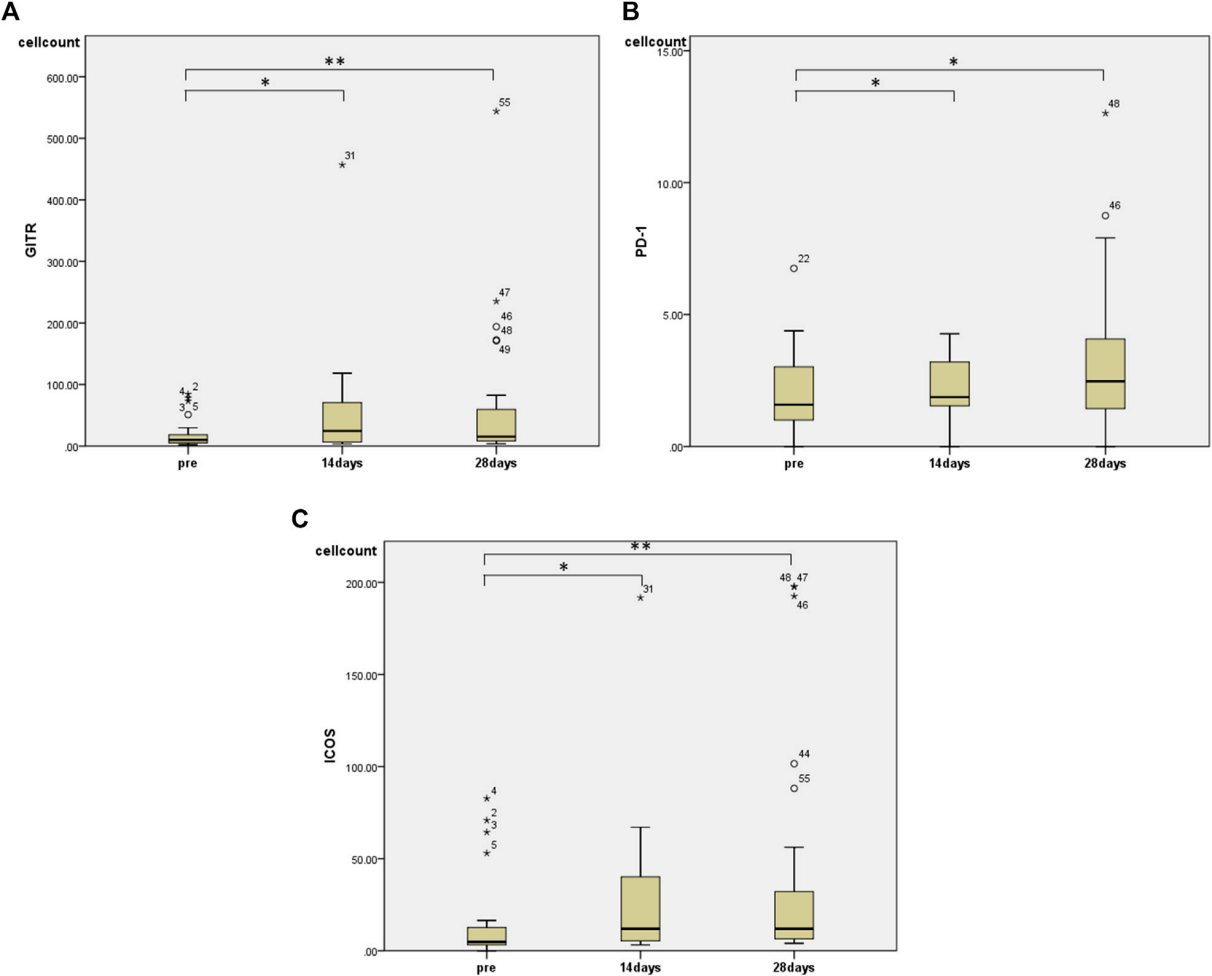
The effects of HET and KKT on PBMC subsets. The significant increase was observed in **(A)** GITR, **(B)**PD-1, and **(C)** ICOS at both 14 and 28 days after administration. * *p* < 0.05; ** *p* < 0.01.

Regarding changes in PBMC subsets, a significant increase in the number of cells was observed in all fractions (total T cells, CD4^+^ T cells, CD8^+^ T cells, and NK cells) except for the number of B cells 28 days after the start of HET ad KKT (Figure 2). There was no significant change in the CD4/CD8 ratio. Of note, there was a significant increase in the number of lymphocytes expressing activating receptors such as NKp46 (median 243, range 74.3–910 before administration; median 513, range 117–1,052 at day 28, *p* < 0.01; Figure 3A), NKp30 (median 61.6, range 9.69–319 before administration; median 85.9, range 12.2–427 at day 28, *p* = 0.001; Figure 3B), and suppressing receptor NKG2A (median 160, range 30.8–496 before administration; median 243, range 40.0–584 at day 28, *p* < 0.01; Figure 3C). There was also an upregulation of T-cell activation markers such as TLR4 (median 34.7, range 4.95–135 before administration; median 55.0, range 20.5–186 at day 28, *p* < 0.01; Figure 3D), OX40 (median 1.07, range 0–11.5 before administration; median 2.73, range 0.526–17.0 at day 28, *p* < 0.05; Figures 3E, 4-1BB (median 12.9, range 1.86–83.3 before administration; median 20.7, range 6.84–92.8 at day 28, *p* < 0.01; Figure 3F) in T cells, which may suggest that naive T cells increased after the start of HET and KKT treatment. Notably, the administration of HET and KKT resulted in changes in GITR (median 10.1, range 2.27–84.3 before administration;; median24.6, range 3.57–457 at day 14, *p* < 0.05; median 15.3, range 3.68–544 at day 28, *p* < 0.01; Figure 4A), ICOS(median 4.79, range 0–82.6 before administration;; median 11.9, range 3.13–191 at day 14, *p* < 0.05; median11.9, range 4.04–198 at day 28, *p* < 0.01; Figure 4B), and PD-1 (median 1.58, range 0–6.75 before administration;; median1.87, range 0–4.27 at day 14, *p* < 0.05; median 2.47, range 0–12.6 at day 28, *p* < 0.05; Figure 4C) on days 14 and 28.

## Discussion

In this study, we found immunological modulation, which has some advantages regarding the prevention of infectious diseases in HCWs who were treated with a combination of HET and KKT, adjusting for comorbidities. The finding that HET and KKT modulate immune cells *in vivo* provides further evidence that Kampo medicines directly or indirectly modulate the immune system. This finding is consistent with the immunomodulatory effects proposed for some of these compounds (Mantani et al., 1999; Hayashi et al., 2007; Wolkerstorfer et al., 2009; Zhou et al., 2013).

Vaccination is certainly a powerful protective approach against infection. However, because the effect of vaccination against mutated viruses is still unknown, and it will take time to produce vaccines in case of the spread of infections caused by other viruses, Kampo medicine may be another option to consider. HET and KKT may be able to prevent infection and serious illness when vaccines cannot be supplied based on the results of this study. Kampo medicine was invented thousands of years ago when infectious diseases were rampant and had gained experience over thousands of years. If we can make use of these Kampo formulas in terms of prevention, then it may be one of the best strategies for treating and managing infectious diseases.

Moreover, “Cytokine storm,” inflammation-mediated severe lung damage, and defective hemostasis are the main underlying causes for morbidity and mortality in COVID-19 patients (Huang et al., 2020). Therefore, infections must be controlled to prevent cytokine storms caused by excessive inflammation. We have already shown that the administration of another Kampo formula, juzentaihoto (JTT), modulates NK cell function. In other words, the administration of JTT activated NK cells, but, at the same time, it suppressed NK cells, thereby regulating NK cell function (Ogawa-Ochiai et al., 2021).

This study also showed that the administration of the HET–KKT combination increased the number of NK cells and T cells, the cytotoxic activity of NK cells, the total number of T cells, and CD_4_/CD_8_-positive cells, indicating that the infection protection ability was enhanced.

NK cells are an essential component of the innate immune system and play a major role in the elimination of virus-infected cells. NK cells express multiple activation receptors such as NKp46 and NKp30 on their cell surface, which are required to recognize specific ligands on potential target cells. By contrast, NKG2A plays an important role in the prevention of excessive inflammation. Of particular interest is the finding of this study that NK cells, mainly the NK subset, which expresses not only NKp46 and NKp30 but also NKG2A receptors on their surface, were significantly increased after the administration of HET and KKT, which may suggest immunomodulation of NK cytotoxicity following treatment with HET and KKT during the COVID-19 pandemic. Recent reports (Liao et al., 2020; Maucourant et al., 2020; Zheng et al., 2020; Bjorkstrom et al., 2021) have showed that NK cells exerted antiviral effects primarily in the lungs early after SARS-CoV-2 infection, that decrease in NK cells was associated with severity of COVID-19, and that expression of activating receptors such as NKp46 and NKp30 on NK cells was associated with antiviral effects, thus suggesting that an increase in the number of circulating NK cells positive for NKp46 and NKp30 may have a protective effect on viral infections such as SARS-CoV-2 in anti-COVID-19 antibody-negative health care workers. However, unexpectedly, in this study, the increase in the number of NKp46 and NKp30-positive circulating lymphocytes after the use of HET / KKT was mainly caused by the increase in the number of NKp46-positive T cells and NKp30-positive T cells. The functions of human T cells that express such activated NK cell receptors, also called a NK-like T cells, are not well understood as well as their roles in COVID-19. Nevertheless, previous studies (Brenchley et al., 2006; Tang et al., 2008; Cupedo et al., 2009; Hudspeth et al., 2012; Hudspeth et al., 2013) have demonstrated that such NK-like T cells are increased by activation stimuli, are endogenous to tonsil tissue, and exert antiviral activity against HIV, thus leading to a hypothesis that an increase in NK-like T cells after the use with HET / KKT in health care workers results in anti-SARS-CoV-2. This hypothesis may be supported by the fact that the function of NKp46 and NKp30 is speculated to be independent of their expressing cells (Tang et al., 2008; Correia et al., 2009; Hudspeth et al., 2013). In addition, in this study, the number of lymphocytes positive for the inhibitory NK receptor NKG2A was also increased in health care workers after using the HET / KKT. A recent report (Zheng et al., 2020) has showed an increase in NKG2A-positive lymphocytes in COVID-19 patients, suggesting that increased NKG2A expression as seen in this study may lead to decreased lymphocyte function in terms of anti-COVID-19. In contrast, it has been reported that upregulation of NKG2A expression in lymphocytes suppresses the excessive immune response of T cells and leads to the maintenance of immunological homeostasis (Jabri et al., 2002). Therefore it remains unclear whether the increase in NKG2A-positive lymphocytes in health care workers after using HET / KKT could act beneficial in preventing COVID-19. These should be clarified in future studies.

There was also an upregulation of T-cell activation markers such as TLR4, OX40, 4-1BB, and TIGIT in T cells, which may suggest that naive T cells increased after the start of HET and KKT treatment. The expression of T-cell activation markers was increased, suggesting an increase in activated T cells as well.

The administration of HET and KKT also promotes T cell-independent activation and interferon production in B cells. This combination of Kampo medicines may be useful for infection defense. On the contrary, along with the promotion of T-cell activation and T-cell differentiation, markers involved in regulation were also significantly increased, indicating immunomodulate effect. Because this was an investigative observational study, we did not have a control group of non-treated or placebo-treated subjects, but a previous study (Nakagami et al., 2019) has shown that there is little inter-individual variation in these markers in placebo-treated subjects.

Since coronaviruses can enter cells in a short time, phagocytes or antibodies alone cannot eliminate the virus. CD8^+^ killer T cells (cytotoxic T cells) are important, as are NK cells, which destroy virus-infected cells and deprive the virus of a place to multiply. CD4^+^ helper T cells are also essential for differentiation of IgG antibody-producing B cells and memory cells (Lucas et al., 2020).

Because the subjects were not infected, the significance of the immunomodulatory functions of HET and KKT during actual infection is not clear. However, the results suggest that they may be effective in preventing infection or severe disease through immunomodulatory changes.

This study had the following limitations. First, there is no evidence that the drug actually increased T cell or NK cell function as only a numerical increase in these immune cells was observed without functional analysis. Second, there is no direct evidence of efficacy of HET and KKT on SARS-CoV-2. Finally, there is no theoretical reason for the increase in T cells or activated NK cells following HET and KKT treatment as no patient was actually infected with coronavirus infection. It is not clear which components contained in these formulas have immune activity, which must be analyzed in the future.

## Conclusion

In conclusion, our findings showed that HET and KKT may prevent the onset of COVID-19 through their immunomodulatory effects. In the future, we would like to clarify the preventive effects of HET and KKT by conducting prospective studies on infected patients.

## Data Availability

The raw data supporting the conclusions of this article will be made available by the authors, without undue reservation.

## References

[B1] Abe S. Tansho S. Ishibashi H. Akagawa G. Komatsu Y. Yamaguchi H. (1999). Protection of Immunosuppressed Mice from Lethal Candida Infection by Oral Administration of a Kampo Medicine, Hochu-Ekki-To. Immunopharmacol Immunotoxicol 21, 331–342. 10.3109/08923979909052766 10319284

[B2] Bjorkstrom N. K. Strunz B. Ljunggren H. G. (2021). Natural Killer Cells in Antiviral Immunity. Nat. Rev. Immunol. 10.1038/s41577-021-00558-3 PMC8194386 34117484

[B3] Brenchley J. M. Price D. A. Schacker T. W. Asher T. E. Silvestri G. Rao S. (2006). Microbial Translocation Is a Cause of Systemic Immune Activation in Chronic HIV Infection. Nat. Med. 12, 1365–1371. 10.1038/nm1511 17115046

[B4] Correia M. P. Cardoso E. M. Pereira C. F. Neves R. Uhrberg M. Arosa F. A. (2009). Hepatocytes and IL-15: a Favorable Microenvironment for T Cell Survival and CD8+ T Cell Differentiation. J. Immunol. 182, 6149–6159. 10.4049/jimmunol.0802470 19414768

[B5] Cupedo T. Crellin N. K. Papazian N. Rombouts E. J. Weijer K. Grogan J. L. (2009). Human Fetal Lymphoid Tissue-Inducer Cells Are Interleukin 17-producing Precursors to RORC+ CD127+ Natural Killer-like Cells. Nat. Immunol. 10, 66–74. 10.1038/ni.1668 19029905

[B6] Gao W. Sanna M. Tsai M. K. Wen C. P. (2020). Geo-temporal Distribution of 1,688 Chinese Healthcare Workers Infected with COVID-19 in Severe Conditions-A Secondary Data Analysis. PLoS One 15, e0233255. 10.1371/journal.pone.0233255 32407411 PMC7224552

[B7] Hayashi K. Imanishi N. Kashiwayama Y. Kawano A. Terasawa K. Shimada Y. (2007). Inhibitory Effect of Cinnamaldehyde, Derived from Cinnamomi Cortex, on the Growth of Influenza A/PR/8 Virus *In Vitro* and *In Vivo* . Antivir. Res 74, 1–8. 10.1016/j.antiviral.2007.01.003 17303260

[B8] Huang C. Wang Y. Li X. Ren L. Zhao J. Hu Y. (2020). Clinical Features of Patients Infected with 2019 Novel Coronavirus in Wuhan, China. Lancet 395, 497–506. 10.1016/S0140-6736(20)30183-5 31986264 PMC7159299

[B9] Hudspeth K. Fogli M. Correia D. V. Mikulak J. Roberto A. Della Bella S. (2012). Engagement of NKp30 on Vδ1 T Cells Induces the Production of CCL3, CCL4, and CCL5 and Suppresses HIV-1 Replication. Blood 119, 4013–4016. 10.1182/blood-2011-11-390153 22403253

[B10] Hudspeth K. Silva-Santos B. Mavilio D. (2013). Natural Cytotoxicity Receptors: Broader Expression Patterns and Functions in Innate and Adaptive Immune Cells. Front. Immunol. 4, 69. 10.3389/fimmu.2013.00069 23518691 PMC3603285

[B11] Jabri B. Selby J. M. Negulescu H. Lee L. Roberts A. I. Beavis A. (2002). TCR Specificity Dictates CD94/NKG2A Expression by Human CTL. Immunity 17, 487–499. 10.1016/s1074-7613(02)00427-2 12387742

[B12] Kluytmans-van den Bergh M. F. Q. Buiting A. G. M. Pas S. D. Bentvelsen R. G. van den Bijllaardt W. van Oudheusden A. J. G. (2020). Prevalence and Clinical Presentation of Health Care Workers with Symptoms of Coronavirus Disease 2019 in 2 Dutch Hospitals during an Early Phase of the Pandemic. JAMA Netw. Open. 3, e209673. 10.1001/jamanetworkopen.2020.9673 32437576 PMC7243090

[B13] Kuroiwa A. Liou S. Yan H. Eshita A. Naitoh S. Nagayama A. (2004). Effect of a Traditional Japanese Herbal Medicine, Hochu-Ekki-To (Bu-Zhong-Yi-Qi Tang), on Immunity in Elderly Persons. Int. Immunopharmacol. 4, 317–324. 10.1016/j.intimp.2003.12.004 14996423

[B14] Liao M. Liu Y. Yuan J. Wen Y. Xu G. Zhao J. (2020). Single-cell Landscape of Bronchoalveolar Immune Cells in Patients with COVID-19. Nat. Med. 26, 842–844. 10.1038/s41591-020-0901-9 32398875

[B15] Lucas C. Wong P. Klein J. Castro T. B. R. Silva J. Sundaram M. (2020). Longitudinal Analyses Reveal Immunological Misfiring in Severe COVID-19. Nature 584, 463–469. 10.1038/s41586-020-2588-y 32717743 PMC7477538

[B16] Mantani N. Andoh T. Kawamata H. Terasawa K. Ochiai H. (1999). Inhibitory Effect of Ephedrae Herba, an oriental Traditional Medicine, on the Growth of Influenza A/PR/8 Virus in MDCK Cells. Antivir. Res 44, 193–200. 10.1016/S0166-3542(99)00067-4 10651070

[B17] Maucourant C. Filipovic I. Ponzetta A. Aleman S. Cornillet M. Hertwig L. (2020). Natural Killer Cell Immunotypes Related to COVID-19 Disease Severity. Sci. Immunol. 5. 10.1126/sciimmunol.abd6832 PMC7665314 32826343

[B18] Mori K. Kido T. Daikuhara H. Sakakibara I. Sakata T. Shimizu K. (1999). Effect of Hochu-Ekki-To (TJ-41), a Japanese Herbal Medicine, on the Survival of Mice Infected with Influenza Virus. Antivir. Res 44, 103–111. 10.1016/S0166-3542(99)00048-0 10669260

[B19] Nakagami Y. Suzuki S. Espinoza J. L. Vu Quang L. Enomoto M. Takasugi S. (2019). Immunomodulatory and Metabolic Changes after Gnetin-C Supplementation in Humans. Nutrients 11, 1403. 10.3390/nu11061403 PMC6628299 31234376

[B20] Ogawa-Ochiai K. Katagiri T. Sato Y. Shirai A. Ishiyama K. Takami A. (2021). Natural Killer Cell Function Changes by the Japanese Kampo Medicine Juzentaihoto in General Fatigue Patients. Adv. Integr. Med. 8, 33–43. 10.1016/j.aimed.2019.12.003

[B21] Satoh N. Sakai S. Kogure T. Tahara E. Origasa H. Shimada Y. (2005). A Randomized Double Blind Placebo-Controlled Clinical Trial of Hochuekkito, a Traditional Herbal Medicine, in the Treatment of Elderly Patients with Weakness N of One and Responder Restricted Design. Phytomedicine 12, 549–554. 10.1016/j.phymed.2004.06.014 16121514

[B22] Tang Q. Grzywacz B. Wang H. Kataria N. Cao Q. Wagner J. E. (2008). Umbilical Cord Blood T Cells Express Multiple Natural Cytotoxicity Receptors after IL-15 Stimulation, but Only NKp30 Is Functional. J. Immunol. 181, 4507–4515. 10.4049/jimmunol.181.7.4507 18802053 PMC2614673

[B23] Tatsumi K. Shinozuka N. Nakayama K. Sekiya N. Kuriyama T. Fukuchi Y. (2009). Hochuekkito Improves Systemic Inflammation and Nutritional Status in Elderly Patients with Chronic Obstructive Pulmonary Disease. J. Am. Geriatr. Soc. 57, 169–170. 10.1111/j.1532-5415.2009.02034.x 19170793

[B24] Tokura Y. Sakurai M. Yagi H. Furukawa F. Takigawa M. (1998). Systemic Administration of Hochu-Ekki-To (Bu-zhong-yi-qi-tang), a Japanese-Chinese Herbal Medicine, Maintains Interferon-Gamma Production by Peripheral Blood Mononuclear Cells in Patients with Mycosis Fungoides. J. Dermatol. 25, 131–133. 10.1111/j.1346-8138.1998.tb02365.x 9563284

[B25] Wang D. Hu B. Hu C. Zhu F. Liu X. Zhang J. (2020). Clinical Characteristics of 138 Hospitalized Patients with 2019 Novel Coronavirus-Infected Pneumonia in Wuhan, China. JAMA 323, 1061–1069. 10.1001/jama.2020.1585 32031570 PMC7042881

[B26] Wolkerstorfer A. Kurz H. Bachhofner N. Szolar O. H. (2009). Glycyrrhizin Inhibits Influenza A Virus Uptake into the Cell. Antivir. Res 83, 171–178. 10.1016/j.antiviral.2009.04.012 19416738 PMC7126985

[B27] Yamaoka Y. Kawakita T. Nomoto K. (2001). Protective Effect of a Traditional Japanese Medicine Hochu-Ekki-To (Chinese Name: Bu-Zhong-Yi-Qi-Tang), on the Susceptibility against Listeria Monocytogenes in Infant Mice. Int. Immunopharmacol. 1, 1669–1677. 10.1016/S1567-5769(01)00076-5 11562059

[B28] Zheng M. Gao Y. Wang G. Song G. Liu S. Sun D. (2020). Functional Exhaustion of Antiviral Lymphocytes in COVID-19 Patients. Cell Mol Immunol 17, 533–535. 10.1038/s41423-020-0402-2 32203188 PMC7091858

[B29] Zhou Y. X. Zhang H. Peng C. (2013). Puerarin: a Review of Pharmacological Effects. Phytother. Res. 28, 961–975. 10.1002/ptr.5083 24339367

